# New perspectives on community-acquired pneumonia in 388 406 patients. Results from a nationwide mandatory performance measurement programme in healthcare quality

**DOI:** 10.1136/thx.2008.109785

**Published:** 2009-05-18

**Authors:** S Ewig, N Birkner, R Strauss, E Schaefer, J Pauletzki, H Bischoff, P Schraeder, T Welte, G Hoeffken

**Affiliations:** 1 Thoraxzentrum Ruhrgebiet, Kliniken für Pneumologie und Infektiologie, Herne und Bochum, Germany; 2 BQS Bundesgeschäftsstelle Qualitätssicherung, Duesseldorf, Germany; 3 Universitaetsklinikum Erlangen, Germany; 4 SRH Kliniken Heidelberg, Germany; 5 Thoraxklinik Heidelberg, Germany; 6 Medizinische Hochschule Hannover, Germany; 7 Universitaetsklinikum Carl Gustav Carus, Fachkrankehaus Coswig, Germany

## Abstract

**Background:**

The database of the German programme for quality in healthcare including data of every hospitalised patient with community-acquired pneumonia (CAP) during a 2-year period (n = 388 406 patients in 2005 and 2006) was analysed.

**Methods:**

End points of the analysis were: (1) incidence; (2) outcome; (3) performance of the CRB-65 (C, mental confusion; R, respiratory rate ⩾30/min; B, systolic blood pressure <90 mm Hg or diastolic blood pressure ⩽60 mm Hg; 65, age ⩾65 years) score in predicting death; and (4) lack of ventilatory support as a possible indicator of treatment restrictions. The CRB-65 score was calculated, resulting in three risk classes (RCs).

**Results:**

The incidence of hospitalised CAP was 2.75 and 2.96 per 1000 inhabitants/year in 2005 and 2006, respectively, higher for males (3.21 vs 2.52), and strongly age related, with an incidence of 7.65 per 1000 inhabitants/year in patients aged ⩾60 years over 2 years. Mortality (13.72% and 14.44%) was higher than reported in previous studies. The CRB-65 RCs accurately predicted death in a three-class pattern (mortality 2.40% in CRB-65 RC 1, 13.43% in CRB-65 RC 2 and 34.39% in CRB-65 RC 3). The first days after admission were consistently associated with the highest risk of death throughout all risk classes. Only a minority of patients who died had received mechanical ventilation during hospitalisation (15.74%).

**Conclusions:**

Hospitalised CAP basically is a condition of the elderly associated with a higher mortality than previously reported. It bears a considerable risk of early mortality, even in low risk patients. CRB-65 is a simple and powerful tool for the assessment of CAP severity. Hospitalised CAP is a frequent terminal event in chronic debilitated patients, and a limitation of treatment escalation is frequently applied.

In the last two decades, considerable progress has been made in the investigation of community-acquired pneumonia (CAP), for example in the understanding of severity assessment, underlying pathogen patterns and the importance of rapid initiation of appropriate empiric antimicrobial treatment. These are reflected in current authoritative guidelines for the management of adult CAP from the USA and Europe.^1^ ^2^ ^3^ ^4^ Nevertheless, only few widely representative data are available reflecting the clinical presentation and outcome of patients with CAP in real life, and only few studies are based on large databases derived from hospital chart records.^5^ ^6^ ^7^

Since 2005, CAP was included in the nationwide mandatory perpetual performance measurement programme in Germany. The National Institute for Quality in Healthcare (BQS) is responsible for inpatient quality assurance in the German healthcare system on behalf of the Federal Joint Committee (Gemeinsamer Bundesausschuss) and the Ministry of Health. BQS together with national specialty groups develops mandatory quality assurance programmes covered by the German Social Code, and collects and analyses data sets of all German hospitals (>2000). The CAP performance programme obligates all hospitals in Germany to document all in-hospital patients with CAP on a predefined quality report sheet.^8^ ^9^ After 2 years, the database from this performance measurement programme offers a unique opportunity to report on the results of all adult patients (⩾18 years) in Germany treated in hospital with CAP in particular regarding epidemiology, attitudes of management and outcomes.

## Methods

### Database

The data items required for the nationwide performance measurement programme were defined by the specialty group on CAP together with the BQS. A case of CAP was identified by encoding pneumonia without severe immunosuppression (HIV infection, solid organ or bone marrow/stem cell transplants, severe neutropenia) as the principal diagnosis of hospital admission. The underlying International Classification of Diseases (ICD-10-German modification (GM)) codes used for inclusion and exclusion of cases are given in Appendix 1 (available online). These codes clearly exclude acute bronchitis and exacerbations of chronic obstructive pulmonary disease (COPD). Patients transferred from another hospital had to have CAP as the initial admission diagnosis to be included. Rehabilitation facilities in Germany are not hospitals, and pneumonia acquired in these facilities was therefore considered to be community acquired. Malignancies were not excluded because they do not represent severe immunosuppression per se, even during treatment, unless there is severe neutropenia, which in turn represents an exclusion criterion. Aspiration pneumonia was included since it is considered as a main cause of CAP.

The database comprised information on the referral mode (from home, nursing home, another hospital or rehabilitation facilities), age and sex, comorbidities according to the ICD-10-GM (optional), clinical condition (chronically bedridden defined as definite inability to undergo mobilisation prior to admission), respiratory rate, systolic and diastolic blood pressure, presence of acute pneumonia-related mental confusion at admission, the use of ventilatory support (non-invasive or invasive), the presence of stability criteria at discharge (including respiratory rate, systolic and diastolic blood pressure, and mental state) and outcome (survival or death). These data were assessed electronically.

The items of the CRB-65 (C, mental confusion; R, respiratory rate ⩾30/min; B, systolic blood pressure <90 mm Hg or diastolic blood pressure ⩽60 mm Hg; 65, age ⩾65 years) score were included in the raw data. Three risk classes were calculated (modified according to Lim *et al*^10^) as follows: risk class (RC) 1 (CRB-65 = 0 points); RC 2 (CRB-65 = 1 or 2 points); RC 3 (CRB-65 = 3 or 4 points or mechanical ventilation at admission, since no reliable assessment of respiratory rate is realistic in these latter patients).

Additional data exclusively retrieved for purposes of the nationwide performance measurement programme were not included in this analysis. No data were available on pathogen patterns and choices of antimicrobial treatment.

### Statistics

Categorial variables were compared using the χ^2^ test and giving the relative risks (RRs) for testing for differences across the two observation years. The significance level was chosen to be 5%. Baseline population figures for the calculation of the incidence of pneumonia originated from the Statististisches Bundesamt.^11^ The incidence was calculated by dividing the number of new cases per year by the number of persons at risk per year, illustrated as number per 1000 inhabitants (aged ⩾18 years) and year.

## Results

### Database

During 2005 and 2006, 1310 of 1435 (91.3%) and 1377 of 1433 hospitals (96.1%), respectively, participated in the performance measurement programme and delivered complete data sets. Overall, 388 406 (2005, 186 691; 2006, 201 715) inpatient data records of patients with CAP were retrieved and analysed.

### Incidence of hospitalised CAP

In 2005 and 2006, the number of inhabitants in Germany was 82 437 995 and 82 314 906, respectively, including 67 880 591 inhabitants in 2005 and 68 072 756 in 2006 aged ⩾18 years (population at risk).^11^ Thus, the incidence of hospitalised CAP was 2.75 and 2.96 per 1000 inhabitants (⩾18 years) and year in 2005 and 2006, respectively.

**Figure 1:**
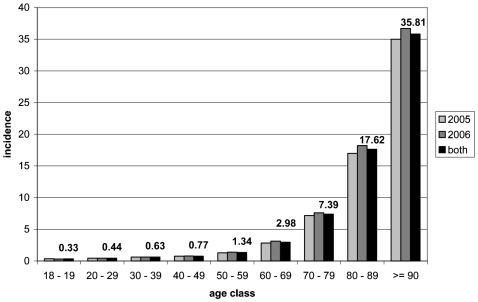
Distribution of the incidence of hospitalised community-acquired pneumonia per 1000 inhabitants and year according to age classes. Incidences in 2005, 2006 and both are shown. The indicated incidence numbers refer to the mean incidence of an age class.

The incidence of hospitalised CAP was strongly age dependent and increased with every decade (fig 1). The mean incidence in persons ⩾60 years was 7.65 per 1000 inhabitants and year over 2 years.

**Figure 2:**
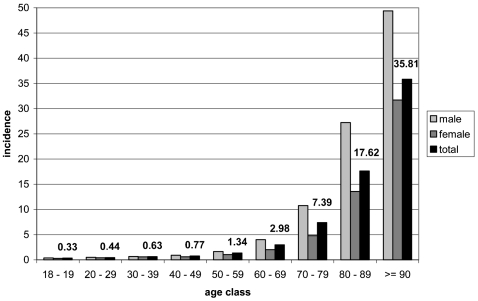
Distribution of incidence of hospitalised community-acquired pneumonia per 1000 inhabitants and year according to age classes. Incidences for sex (overall 2005 and 2006) compared with overall incidence are given. The indicated incidence numbers refer to the mean incidence of an age class.

The mean incidence for males was 3.21 per 1000 inhabitants per year as compared with 2.52 for females (fig 2)

### Patient characteristics

#### Age and sex

In both years, the age distribution was remarkably similar, with a median of 76 years (interquartile range (IQR) = 20 and 19 years in 2005 and 2006, respectively; mean age = 71.94 and 72.09 years, SD = 16.86 and 17.00). The large majority of patients were aged ⩾60 years, accounting for 81.00% of patients. The single largest age decade including 28.64% of patients was 80–89 years old (table 1).

**Table 1:** Age distribution

Age class (years)	2005, n (%)	2006, n (%)	Total, n (%)
18–19	674 (0.36)	626 (0.31)	1300 (0.33)
20–29	4321 (2.32)	4329 (2.15)	8650 (2.23)
30–39	7290 (3.91)	7071 (3.51)	14 361 (3.70)
40–49	10 213 (5.47)	10 712 (5.31)	20 925 (5.39)
50–59	13 448 (7.21)	15 094 (7.48)	28 542 (7.35)
60–69	28 380 (15.21)	30 569 (15.15)	58 949 (15.18)
70–79	48 904 (26.20)	53 498 (26.52)	102 402 (26.37)
80–89	52 258 (28.00)	58 965 (29.23)	111 223 (28.64)
⩾90	21 157 (11.34)	20 849 (10.34)	42 006 (10.82)
Total patients with valid data	186 645	201 713	388 358

Forty-eight cases of the total sample had no valid age data.

**Figure 3:**
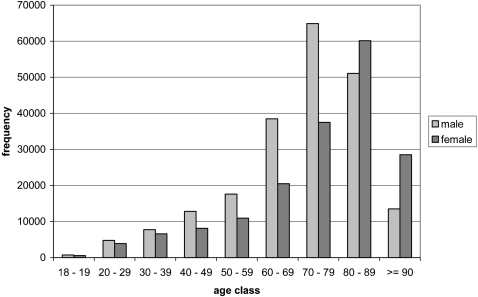
Age and sex distribution of patients hospitalised with community-acquired pneumonia (total population in 2005 and 2006).

There was a preponderance of male patients in both years (211 579 (54.47%) male vs 176 827 (45.53%) female). The raw number of male patients exceeded the number of female patients until the seventh decade (70–79); thereafter more female than male patients were documented (fig 3).

#### Admission to hospital

A total of 86 387 patients were admitted from nursing homes and 16 158 from other hospitals or rehabilitation facilities.

#### Comorbidities and clinical condition

Patient comorbidity according to ICD-10-GM is given in table 2. The most frequent comorbidity was cardiac comorbidity (19.2%), followed by central nervous system disorders (13.8%), pulmonary comorbidity other than COPD (13.0%), and diabetes mellitus (11.9%).

**Table 2:** Patient comorbidity

Comorbidity	2005	2006	Total
	n (% of 2005 population)	n (% of 2006 population)	n (% of overall population)
Cardiac comorbidity	36 380 (19.49)	38 307 (18.99)	74 687 (19.23)
CNS disorders	25 660 (13.74)	27 763 (13.76)	53 423 (13.75)
Pulmonary diseases (other than COPD)	23 506 (12.59)	27 171 (13.47)	50 677 (13.05)
Diabetes mellitus	22 189 (11.89)	23 991 (11.89)	46 180 (11.89)
COPD	17 247 (9.24)	19 314 (9.57)	36 561 (9.41)
Renal diseases	15 536 (8.32)	18 034 (8.94)	33 570 (8.64)
Dementia	11 037 (5.91)	12 129 (6.01)	23 166 (5.96)
Malignancy (other than bronchial)	5805 (3.11)	6852 (3.40)	12 657 (3.26)
Lung cancer	2645 (1.42)	3427 (1.70)	6072 (1.56)
Liver diseases	2921 (1.56)	2948 (1.46)	5869 (1.51)
Total	100 381 (53.77)	109 616 (54.34)	209 997 (54.07)

Listed are the 10 most frequently recorded comorbidities.

Comorbidity as defined was present in 209 997 patients (54.07%). Some patients had more than one comorbidity. Of patients with at least one comorbidity, 36 604 (17.43%) died.

CNS, central nervous system; COPD, chronic obstructive pulmonary disease.

Overall, 26.07% (101 249/388 406) patients were classified as chronically bedridden.

#### Pneumonia severity at admission

Of the whole CAP population, 16.55% of patients belonged to CRB-65 RC 1, 71.55% to CRB-65 RC 2 and 11.91% to CRB-65 RC 3 (table 3).

**Table 3:** Pneumonia severity at admission according to the CRB-65 score

CRB-65 risk class	2005, n (%)	2006, n (%)	Total, n (%)
1 (CRB-65 = 0)	30 285 (16.22)	33 982 (16.85)	64 267 (16.55)
2 (CRB-65 = 1, 2)	127 652 (68.38)	150 238 (74.48)	277 890 (71.55)
3 (CRB-65 = 3, 4)	28 754 (15.40)	17 495 (8.67)	46 249 (11.91)
Total patients with valid data (n)	186 691	201 715	388 406

The CRB-65 risk score is based on four parameters: C, mental confusion; R, respiratory rate ⩾30/min; B, systolic blood pressure <90 mm Hg or diastolic blood pressure ⩽60 mmHg; 65, age ⩾65 years.

One point is assigned in the case of the presence of each parameter, adding up to a minimum of zero and a maximum of four points. The CRB-65 risk score classification is then calculated as follows: CRB-65, 0 points equivalent to risk class 1; CRB-65, 1 or 2 points equivalent to risk class 2; CRB-65, 3 or 4 points or mechanical ventilation at admission equivalent to risk class 3.

Overall, 3924 (2.10%) and 4428 (2.20%) patients received ventilator support at admission in 2005 and 2006, respectively (p = 0.045, χ^2^ test). A total of 10 294 (5.51%) patients with CAP received ventilator support at admission or during hospitalisation in 2005 and 12 106 (6.00%) in 2006 (p<0.001, χ^2^ test).

#### Mortality

Overall in-hospital mortality was 13.72% (95% CI 13.57% to 13.88%, n = 25 622) in 2005 and 14.44% (95% CI 14.29% to 14.60%, n = 29 132) in 2006 (p<0.001, χ^2^ test). Mortality varied within hospitals from 0% to 40% and from 0% to 35% in 2005 and 2006, respectively.

**Figure 4:**
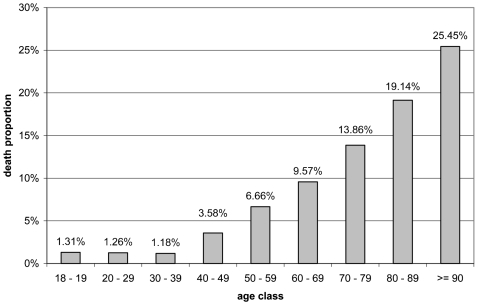
Distribution of in-hospital death proportions of patients hospitalised with community-acquired pneumonia according to age classes (total population in 2005 and 2006).

In patients older than 40 years, mortality increased with age (fig 4). There was no statistically significant difference in mortality between men and women (overall RR = 1.01; 95% CI 0.99 to 1.02.

Patients admitted from nursing homes had a statistically significantly higher mortality (2005, 10 169/30 601, 24.94%; 2006, 11 674/33 943, 25.59%) compared with patients from other provenances (2005, 15 453/130 468, 10.59%; 2006, 17 458/138 640, 11.18%) (RR = 2.32; 95% CI 2.29 to 2.36). Accordingly, chronically bedridden patients had a high mortality of 30.37% (30 745/101 249; RR = 3.63; 95% CI 3.58 to 3.69). On the other hand, excluding patients from nursing homes and those who were chronically bedridden reduced the overall mortality to 7.60% (19 842/261 038).

#### Mortality and comorbidities

In patients with defined comorbidities, the highest mortality was observed in patients with malignant diseases (28.20% for patients with tumours other than bronchial tumours, 25.21% for patients with bronchial tumours). Pulmonary comorbidities excluding COPD and dementia were also associated with a high mortality of 24.45% and 22.36%, respectively, whereas patients with COPD had a remarkably lower mortality of 10.12% (table 4).

**Table 4:** Patient mortality according to comorbidity

Comorbidity	2005 deaths (%)	2006 deaths (%)	Total (%)
Malignancy (other than bronchial)	27.65	28.66	28.20
Lung cancer	25.07	25.33	25.21
Pulmonary diseases (other than COPD)	24.03	24.82	24.45
Dementia	21.93	22.76	22.36
Renal diseases	20.20	21.30	20.79
CNS disorders	19.27	19.54	19.41
Cardiac comorbidity	17.06	17.63	17.35
Diabetes mellitus	13.41	13.88	13.66
Liver diseases	12.26	13.60	12.93
COPD	9.85	10.37	10.12
Total	17.08	17.75	17.43
No comorbidity	12.50	13.35	12.95

Listed are the 10 most frequently recorded comorbidities.

Comorbidity as defined was present in 209 997 patients. Of these, 36 604 (17.43%) died.

CNS, central nervous system; COPD, chronic obstructive pulmonary disease.

**Figure 5:**
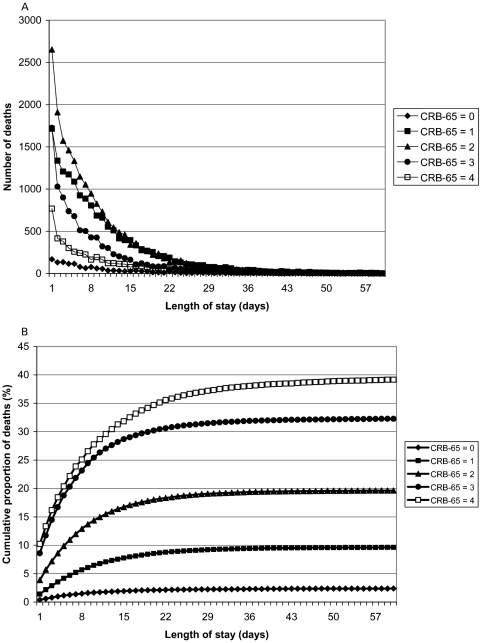
(A) Crude number of deaths during hospitalisation, stratified for CRB-65 (C, mental confusion; R, respiratory rate ⩾30/min; B, systolic blood pressure <90 mm Hg or diastolic blood pressure ⩽60 mm Hg; 65, age ⩾65 years) scores. Data refer to the whole population in 2005 and 2006. Day 0 was not calculated because it is not a complete day. (B) Cumulative proportion of deaths, stratified for CRB-65 scores. Note that the highest mortality is observed during the first days in all risk classes, even in the lowest (CRB-65 = 0). Day 0 was not calculated because it is not a complete day.

In 2005, non-survivors died at day day 5 (median, IQR 9 days) after hospital admission, in 2006 at day 6 (median, IQR 10 days), respectively. However, most patients died within the first days after hospitalisation (fig 5A). This pattern of mortality was consistently present throughout CRB-65 scores, even in the lowest CRB-65 class (fig 5B).

Of those patients who died during hospital stay, only 15.74% (8618/54 754) had received ventilatory support at admission or during hospitalisation (2005, 15.47% (3964/25 622); 2006, 15.98% (4654/29 132)). On the other hand, of those who received ventilator support, 38.47% (8618/22 400) died (2005, 38.51% (3964/10 294); 2006, 38.44% (4654/12 106)). The proportion of mechanically ventilated patients increased steadily with age until the seventh age decade to 31.44%, but decreased to 23.05% and 4.21% in the eighth and ninth decade, respectively.

The proportions of mortality according to the three factors associated with highest mortality cross-referenced with each other are listed in table 5. These increased with every additional factor from 25.29% to 42.79%.

**Table 5:** Proportion of mortality in patients with factors associated with the highest mortality: admission from nursing home, bedridden status and mechanical (non-invasive and invasive) ventilation

Factor	2005, n (%)	2006, n (%)	Total, n (%)
Admission from nursing home and bedridden	8264 (28.96)	9412 (29.66)	17 676 (29.33)
Admission from nursing home and ventilated	478 (39.54)	640 (40.35)	1118 (40.00)
Admission from nursing home, bedridden and ventilated	348 (42.08)	453 (43.35)	801 (42.79)

#### Performance of CRB-65 severity score

The CRB-65 severity score predicted death in a three-class pattern, with overall mortality of 2.40% in CRB-65 RC 1, 13.43% in CRB-65 RC 2 and 34.39% in CRB-65 RC 3 (table 6).

**Table 6:** Performance of CRB-65 severity score to predict in-hospital mortality from community-acquired pneumonia

CRB-65 class (risk class)	2005, n deaths (% of risk class)	2006, n deaths (% of risk class)	Total, n deaths (% of risk class)
RC 1 (CRB-65 = 0)	532 (1.76)	1009 (2.97)	1541 (2.40)
RC 2 (CRB-65 = 1 or 2)	15 439 (12.09)	21 871 (14.56)	37 310 (13.43)
RC 3 (CRB-65 = 3 or 4 or requirement for mechanical ventilation at admission)	9651 (33.56)	6252 (35.76)	15 903 (34.39)
Total deaths	25 622 (13.72)	29 132 (14.44)	54 757 (14.10)

The CRB-65 severity score is based on four parameters: C, mental confusion; R, respiratory rate ⩾30/min; B, systolic blood pressure <90 mm Hg or diastolic blood pressure ⩽60 mm Hg; 65, age ⩾65 years).

RC, risk class.

The length of hospital stay (was associated with the severity of pneumonia (mean length of stay 9.45 vs 12.39 vs 14.50 days (SD: 7.82, 8.47, 10.69) for RC 1–3, respectively, excluding deaths).

## Discussion

The main results of the present analysis of this German nationwide quality performance data on hospitalised patients with CAP are the following:

- The incidence of hospitalised CAP was high, strongly age related and increased with every decade until the eighth decade.
- Mortality was higher than reported in previous studies of selected patients.
- The first days of admission were associated with the highest mortality, and this was consistently true throughout all CRB-65 risk classes.
- The CRB-65 score accurately predicted death in a three-class pattern.
- Only a minority of patients who died had received mechanical ventilation during hospitalisation, hinting at a limitation of treatment escalation for debilitated patients.

Previous epidemiological studies have reported a total incidence of pneumonia including outpatient and inpatient cases of about 1.6–12.0 per 1000 inhabitants and year.^12^ ^13^ ^14^ In a population-based study from a region of Barcelona, Spain, an incidence of 1.62 was found.^12^ Similar studies from Kuopio in Finland and from Elche in Spain found a higher incidence of 11.6 per 1000 inhabitants and year and 12 per 10000 person-years, respectively.^13^ ^14^ In a 1-year study of adult hospitalised CAP in patients residing in two counties in Ohio, USA, the overall incidence was 2.67 per 1000 inhabitants and year.^15^ This latter figure fits perfectly with the incidence found in our database. We also could confirm the higher incidence of CAP in males.

In line with previous epidemiological data, the incidence of CAP was strongly age related. Moreover, a very high number of patients resided in nursing homes (22%) and were chronically bedridden (26%). The highest incidence was reached in the very old (80 years and older). Although these numbers are lower than those reported in a recent large American study reaching 18.3 per 1000 population ⩾65 years,^6^ the estimated additional future healthcare burden is impressive. Given the expected increase in the elderly population in Germany (60 years and older) up to 2050,^11^ another 30 000–60 000 CAP cases will require hospitalisation per year. Similar numbers are to be expected in other ageing European and North American countries.^16^ ^17^ Any future estimation of the healthcare burden of CAP will have to realise that CAP essentially is mainly a condition of the elderly.

Most surveys including patients with hospitalised CAP reported a mortality of ⩽10%.^5^ ^6^ ^7^ ^12^ ^17^ ^18^ ^19^ In this analysis, mortality was considerably higher. It seems reasonable to assume that this difference can be explained mainly by the exclusion of many high-risk patients in most clinical studies. Accordingly, mortality in our analysis for patients admitted from nursing homes was similar to the figures provided for healthcare-related pneumonia (HCAP) in two recent studies (19.8% vs 10%^20^ and 24.6 vs 9.1%^21^ as compared with 24.94% vs 11.18% in our study).

When looking at the time of death in non-survivors, there was a striking excess risk of death in the first 7 days, with the highest proportions during the first day after admission. This is in line with a previous report in patients aged ⩾65 years,^6^ and fits well with many studies indicating that the rapid initiation of appropriate antimicrobial treatment is crucial for the outcome.^22^ ^23^ Moreover, this trend was consistently evident throughout all CRB-65 risk classes, implying that even patients at lowest risk for lethal outcome are at their relatively highest risk mainly within the first days after admission. Interestingly, others could demonstrate that the distribution of hospital deaths over time is the same for the subgroup managed aggressively.^6^

All studies assessing severity assessment scores have unanimously reported a substantial number of patients hospitalised despite a low risk of death according to the score evaluated.^24^ ^25^ ^26^ Although we do not have any information on the reasons for hospitalisation of a large proportion of patients at low risk according to CRB-65 severity assessment in the database underlying this analysis (16–17%), the finding of an increased risk of early death across all CRB-65 classes supports the clinical notion that increased clinical attention is mandatory at least during the first 2 days after admission.

European authors were able to show that simple scores such as the CURB, CURB-65 or CRB-65 score are at least not inferior to the more complex PSI (pneumonia severity index) score.^26^ ^27^ ^28^ Our data show that the simple CRB-65 score which is based exclusively on three clinical signs and age predicts death from CAP in a three-class pattern. The results are comparable with those of the PSI score that predicts death in the range of 0.1–3% in risk classes I–III, 8–13% in class IV and 29–31% in class V.^5^ Although the database underlying this analysis does not allow a comparison of CRB-65 and PSI in the same patients, the performance of CRB-65 presented here adds strong evidence to the usefulness of this score in the assessment of pneumonia severity in real life and emphasises the preference for this simple score as recently advocated.^29^

An intriguing finding was the low number of patients who died having previously received ventilatory support. Although there are no nationwide recognised standard criteria for ventilatory support, it can be regarded as a surrogate parameter for intensive or at least intermediate care. The low proportion of ventilated patients may therefore hint at a considerable number of patients in whom a decision against ICU admission was made. This view is supported by the decreasing proportions of mechanical ventilation in the eighth and ninth age decade. Our findings are in contrast to American data reporting admission to ICU in 22.4% of cases aged ⩾65 years and mechanical ventilation in 7.2%.^6^

The current guidelines on the management of adult CAP do not mention the issue of care for patients with CAP as a terminal event.^1^ ^2^ ^3^ ^4^ This is true despite a traditional notion of CAP as “the old man’s friend”, and despite some recent data reporting a high frequency of “do not resuscitate” (DNR) orders (22%) in patients with hospitalised CAP.^30^ The ignorance regarding CAP frequently representing a terminal disease no longer seems acceptable. In selected chronically debilitated patients it may be more appropriate for them to receive treatment under the special considerations of terminal care. However, such considerations must not rely exclusively on decisions to withhold antimicrobial treatment^31^ or on DNR orders,^30^ but there should be awareness of a range of potentially applicable restrictions.

It might be argued that the population studied may have included an uncertain proportion of patients with HCAP with specific aetiological and microbial resistance patterns bearing an excess mortality beyond age and nursing home pneumonia.^20^ ^21^ ^32^ In our view, the presence, incidence and characteristics of HCAP directly reflect national and regional structures and policies of healthcare in the chronically ill.^33^ Thus, it seems premature and potentially misleading to apply the concept of HCAP in an indiscriminate way.

The main limitation of the present analysis and its underlying database is the case definition of CAP. Since a case of CAP was identified encoding pneumonia as the principal cause of hospital admission, some cases may have been missed, mainly in the case of CAP with severe sepsis or septic shock when the latter was encoded as the principal cause of hospital admission. On the other hand, according to the imprecision of the ICD-10 code (IDC-10-GM), it was not possible to exclude all cases with nosocomial pneumonia. However, this would concern at most 4% of the whole data set. Comparable previous large studies have also been based on ICD codes.^6^ ^7^ We were not able to validate the database externally. However, biases from data validity are estimated to be limited since the data evaluated here mainly included patient characteristics and most simple clinical baseline data. This does not fully apply to the reporting of comorbidity, and these data must be regarded with caution. A nationwide study, on the other hand, with the inclusion of all patients hospitalised with CAP—that is, virtually no selection bias—is unique.

This analysis allows several conclusions. Hospitalised CAP is mainly a condition of the elderly. Hospitalised CAP is a condition with a considerable risk of early mortality after admission, requiring rapid decisions about treatment settings and modalities. It may be worth also giving special attention to patients at low risk during this time frame. CRB-65 is a simple and powerful tool for the assessment of CAP severity which may be of help in routine clinical decision making. Obviously, CAP is a frequent terminal event in chronic debilitated patients, and a limitation of treatment escalation is frequently applied.
